# Zuojin Pill enhances gastrointestinal motility by modulating the pacemaker potentials in interstitial cells of Cajal through multiple signaling pathways

**DOI:** 10.7150/ijms.103507

**Published:** 2024-10-28

**Authors:** Jueun Lee, Seok Jae Ko, Na Ri Choi, Woo-Gyun Choi, Mujin Seo, Seung Hyeon Koo, Daehwa Jung, Min Jae Lee, Jong Hwan Lee, Jae-Woo Park, Byung Joo Kim

**Affiliations:** 1 Department of Longevity and Biofunctional Medicine, School of Korean Medicine, Pusan National University, Yangsan 50612, Republic of Korea.; 2 Department of Clinical Korean Medicine, Graduate School of Kyung Hee University, Seoul 02447, Republic of Korea.; 3 Department of Gastroenterology, College of Korean Medicine, Kyung Hee University, Seoul 02447, Republic of Korea.; 4 Department of Korean Medical Science, Pusan National University School of Korean Medicine, Yangsan, Republic of Korea; 5 Department of Pharmaceutical Engineering, Daegu Hanny University, Gyeongsan 38610, Republic of Korea.; 6 College of Veterinary Medicine, Kangwon National University, Chuncheon 24341, Republic of Korea.; 7 Department of Biomedical Engineering, Dong-Eui University College of Engineering, Busan 47340, Republic of Korea.

**Keywords:** Zuojin Pill, Gastrointestinal Disorders, Interstitial Cells of Cajal, Pacemaker Potential, Intestinal Transit Rate

## Abstract

Zuojin Pill (ZJP) is a traditional herbal preparation used to treat various gastrointestinal (GI) disorders. The purpose of this study was to elucidate the underlying cellular and molecular mechanisms by evaluating the effects of ZJP on the pacemaker activity of isolated interstitial cells of Cajal (ICCs) and on *in vivo* GI motility in mice. We isolated ICCs from mouse small intestine and measured pacemaker potentials by whole-cell patch clamping as well as intracellular calcium signaling by microfluorometry. Intestinal transit rate (ITR) was measured by Evans Blue migration. Administration of ZJP depolarized ICCs and reduced the amplitude and frequency of pacemaker potentials. Pretreatment with the 5-HT4 receptor antagonist RS39604, administration of the muscarinic M3 receptor antagonist 4-DAMP, or intracellular perfusion of the G-protein inhibitor GDP-β-S blocked ZJP-induced ICCs depolarization. In addition, external calcium-free medium and administration of the Ca^2+^-ATPase inhibitor thapsigargin, which depletes intracellular calcium stores, also blocked ZJP-induced ICCs depolarization. Moreover, ZJP-induced ICCs depolarization was inhibited in the presence of the phospholipase C (PLC) inhibitor U-73122, IP_3_-dependent Ca^2+^ release inhibitor xestospongin C, or various mitogen-activated protein kinase (MAPK) inhibitors. Alternatively, ZJP-induced ICCs depolarization in the presence of protein kinase C (PKC) inhibitors. Furthermore, ZJP reversed the reduction of ITR caused by loperamide (Imodium) and normalized the ITR abnormality of two etiologically distinct GI motility disorder (GMD) mouse models. Finally, ZJP increased serum concentrations of the pro-peristalsis factors motilin and substance P. Our results suggest that ZJP depolarizes ICCs via 5-HT4 or muscarinic M3 receptor activation and G-protein dependent calcium-, PLC-, inositol triphosphate-, and MAPK signaling pathways (but not PKC-dependent pathways), leading to enhanced GI motility.

## Introduction

Zuojin Pill (ZJP) is a mixture of the medicinal herbs Coptidis rhizoma and Evodiae fructus long used by practitioners of traditional Chinese medicine (TCM) to treat inflammation, gastrointestinal (GI) disorders, and depression among other ailments 1,2. Controlled studies report that ZJP inhibits the expression and release of pro-inflammatory mediators from macrophages 3 and mitigates depressive symptoms by regulating multiple neurotransmitter systems 4-6. Further, ZJP induces cancer cell death by activating the mitochondrial apoptosis pathway 1,7,8 but conversely exerts hepatoprotective effects 9. The most common indications for ZJP, however, are GI disorders. Zuojin Pill alleviates colitis by regulating the gut microbiota and reduces stomach inflammation via mitogen-activated protein kinase (MAPK) signaling 10,11, helps to mitigate GI dysfunction caused by stress via the TPH2/5-HT pathway 6, and alleviates chronic atrophic gastritis from various causes by normalizing gastric epithelial cell function through TGF-beta1/PI3K/Akt pathway activation 12-14. In addition, ZJP has been used effectively to treat gastritis, excessive stomach acid, and indigestion 1,2.

Proper digestion of food and absorption of nutrients is dependent on efficient GI motility, which involves a precisely coordinated series of smooth muscle contractions and relaxations in the stomach and intestines (peristalsis) 15. Impaired GI motility can cause indigestion, constipation, and diarrhea among other conditions 15, and GI motility disorder (GMD) is one of the most common functional ailments of the GI tract. Functional dyspepsia is the most common GMD, characterized by heartburn, abdominal bloating, indigestion, belching, nausea, and poor GI motility 16,17. Chronic GMD markedly impairs quality of life and daily productivity, and increases healthcare costs 16,17. GI motility is regulated by the enteric and autonomic nervous systems, local cells within the GI tract, and by various circulating hormones 18. Among local regulators are the interstitial cells of Cajal (ICCs), which are critical pacemaker cells controlling the rate of GI motility 15,19,20. These ICCs mediate signal transmission between the autonomic nervous system and smooth muscle cells, thereby maintaining GI peristalsis 19,20. These cells are present throughout the GI tract, and abnormalities can lead to various GMDs 19-21. Understanding ICCs function and dysfunction is therefore essential for comprehending normal GI motility and GMDs. Given the broad spectrum of therapeutic effects on the GI tract attributed to ZJP, it is highly possible that this preparation influences ICCs function, but to date there have been no studies on the regulation of ICCs by ZJP. Therefore, this study aimed to describe the effects of ZJP on ICCs pacemaker activity, the associated signaling pathways, and how these cellular effects influence GI motility.

## Materials and methods

### Preparation of ZJP

The ZJP sample used for all experiments was prepared by decocting 240 g of Coptidis rhizoma and 40 g of Evodiae fructus with 3,000 mL of distilled water in an herbal medicine decoction machine (Coptidis rhizoma Daewoong DW-290, Seoul, Korea) for 2 h. The resulting extract was centrifuged at 3,000 rpm for 10 min (Vision, Daejeon, Korea) and the supernatant obtained was concentrated using a rotary vacuum evaporator (Vision, Daejeon, Korea). Subsequently, the raw concentrate was frozen at -84°C for 24 h in a deep freezer (Fisher Scientific, Pittsburgh, PA, USA) and then freeze-dried using a freeze dryer (Ilshin, Dongducheon, Korea), yielding 33.86 g of dried powder (approximately 14% yield). The dried powder was dissolved in ethanol to achieve the required concentration for experimental use.

### Component analysis of ZJP using ultra-performance liquid chromatography (UPLC)

**Figure 1:**
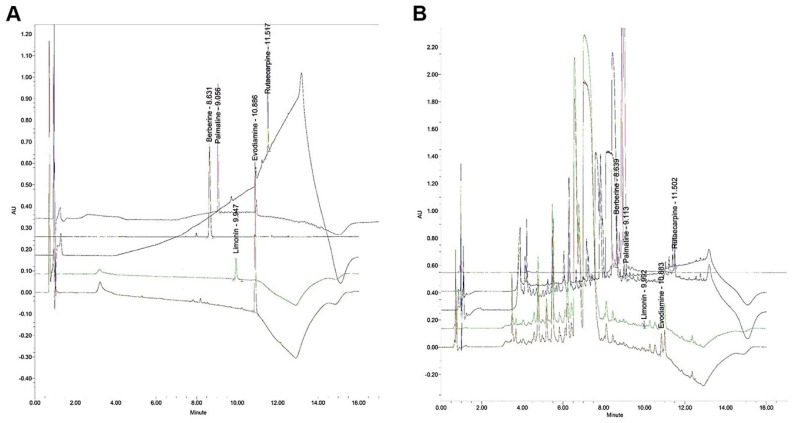
Chromatographic analyses of evodiamine, limonin, rutaecarpine, berberine, and palmatine in the ZJP sample. (A) UPLC chromatogram of the standard. (B) UPLC chromatogram of the five major compounds in ZJP. Palmatine was detected at 254 nm, evodiamine and rutaecarpine at 270 nm, limonin at 280 nm, and berberine 350 nm. UPLC: ultra-performance liquid chromatography.

Bioactive components of the ZJP sample were quantified using an ACQUITYTM ultra-performance LC system (Waters, Milford, MA, USA). The reagents used were methanol (Junsei, Tokyo, Japan), acetonitrile (JT-BAKER, Phillipsburg, NJ, USA), and tertiary-distilled water. Evodiamine and limonin standards were purchase from Sigma (St. Louis, MO, USA) and berverine, palmatine, and rutaecarpine standards were purchased from ChemFaces (Wuhan, China). Berberine and palmatine are derived from Coptis rhizome while evodiamine, limonin, and rutaecarpine are derived from Evodia fruit. Evodiamine and rutaecarpine peaks were detected at 270 nm, limonin at 280 nm, berberine at 350 nm, and palmatine at 254 nm. Two microliters (0.4 mL/min) of sample were injected for each analysis (Tables 1 and 2; Figure 1).

### Isolation of maintenance of ICCs

The small intestine was excised from ICR mice (5-7 days old; 51 males and 48 females; Samtako, Gyeonggi, Republic of Korea) and the mucosa removed. Subsequently, ICCs and other mucosal cells were dissociated by treatment with buffer containing 1.5 mg/mL collagenase (Worthington Biochemical Corporation, Lakewood, NJ, USA), 2.1 mg/mL bovine serum albumin (Sigma-Aldrich; St Louis, MO, USA), 1.8 mg/mL Trypsin inhibitor (Sigma-Aldrich; St Louis, MO, USA) and 0.3 mg/mL ATP (Sigma-Aldrich; St Louis, MO, USA) for 15 min. The cells were then cultured in smooth muscle growth medium (Clonetics, San Diego, CA, USA) at 37°C. Cultured ICCs were used for experiments within 12 h of isolation. In culture, ICCs were morphologically distinct from all other mucosal cells and so were identified for experiments using phase-contrast microscopy. The animal study protocol was approved by the Institutional Animal Care and Use Committee at Pusan National University (Busan, Korea; approval no. PNU-2023-0315). All mice used in this study were housed in a specific pathogen free environment under controlled temperature (21°C-23°C) and humidity (50%-60%) with free access to standard food and water.

### Electrophysiological experiments

Pacemaker potentials of ICCs were recorded at 30°C-33°C in the whole-cell patch clamp configuration. The pipette solution consisted of (in mM) KCl 140, MgCl_2_ 5, K_2_ATP 2.7, NaGTP 0.1, creatine phosphate disodium 2.5, HEPES 5, and EGTA 0.1, and cells were superfused with saline containing (in mM) KCl 5, NaCl 135, CaCl_2_ 2, glucose 10, MgCl_2_ 1.2, and HEPES 10. All recordings were obtained using an Axopatch 200B amplifier (Axon Instruments, Foster City, CA, USA). The pipette resistance was maintained at 3-5 MΩ, and the seal resistance was over 1 GΩ for all recordings saved for analysis. Data were analyzed using pClamp and Origin software version 2018.

### Measurement of the intracellular calcium concentration

ICCs were loaded with the calcium-sensitive fluorescent dye fura-2/AM for 15 min at room temperature and changes in the emission ratio from 340 and 380 nm excitation measured as an estimate of intracellular calcium concentration in response to the indicated treatments using a PTI Delta scan illuminator (Photon Technology International Inc., Birmingham, NJ, USA).

### Intestinal transit rate (ITR) measurement

A total of 43 ICR mice (all males; 5 to 6 weeks old; Samtako, Gyeonggi, Republic of Korea) were used for the ITR experiments. Mice were administered ZJP or vehicle via gavage needle, followed 30 min later by intragastric administration of Evans Blue (5% in saline). The animals were euthanized after another 30 min, and ITR measured as Evans Blue migration distance from the pylorus as a percentage of total small intestine length.

### GMD mouse models

We created two mouse models of GMD using acetic acid (AA) and streptozotocin (STZ). The STZ-induced diabetes model exhibits dysmotility, and major GI complications are associated with diabetes. In this condition, the function of the muscles and nerves in the GI tract is impaired, making normal digestion and bowel movements difficult. The AA-induced GMD model is frequently used to study GI inflammation and dysfunction. AA induces inflammation in the gut, disrupting normal GI motility and leading to dysmotility. To create the AA GMD model, AA (0.5% w/v in saline) was injected via the intraperitoneal route into the abdominal cavity to induce peritoneal irritation. The STZ diabetes model was also established because these mice demonstrate comorbid dysmotility due to smooth muscle and peripheral nerve dysfunction. Briefly, male ICR mice (4-5 weeks) were injected with STZ (200 mg/kg; Sigma-Aldrich). Two months later, blood was collected from the tail vein and blood glucose concentration was measured using the ONETOUCH kit (Johnson & Johnson Medical Company, New Brunswick, NJ, USA). Only mice with blood glucose levels >16 mM/L were considered diabetic. All STZ-induced diabetes model mice demonstrated GMD. The ITR was measured in both models using the same Evan blue migration method.

### Gut hormone levels

25 ICR mice (all males; 5-6 weeks old; Samtako, Gyeonggi, Republic of Korea) were used for intestinal hormone measurements. Mice received ZJP (0.5 g/kg) or vehicle once a day for 5 days. Changes in motilin (MTL), substance P (SP), somatostatin (SS), and vasoactive intestinal polypeptide (VIP) were measured in serum samples by Abbkine Scientific Co., Ltd. (Atlanta, GA, USA).

### Drugs

Thapsigargin (Cat. No. 1138), Y25130 (0380), RS39604 (0991), SB269970 (1612), xestospongin C (1280), PD98059 (1213), SB203580 (1402), SP600125 (1496), and loperamide (0840) were purchased from Tocris Bioscience (Bristol, United Kingdom), while methoctramine (104807-46-7), 4-DAMP (1952-15-4), guanosine 5'‑O‑(2‑thiodiphosphate) (GDP‑β‑S; 97952-36-8), U-73312 (112648-68-7), staurosporine (62996-74-1), go6976 (136194-77-9), and rottlerin (82-08-6) were obtained from Sigma‑Aldrich (St Louis, MO, USA).

### Statistical analysis

All results are expressed as mean ± standard error of the mean (SE). Group differences were evaluated by one-way analysis of variance or Student's t-test. A p <0.05 was considered statistically significant for all tests.

## Results

### ZJP depolarizes ICCs and reduces the amplitude and frequency of pacemaker potentials

**Figure 2:**
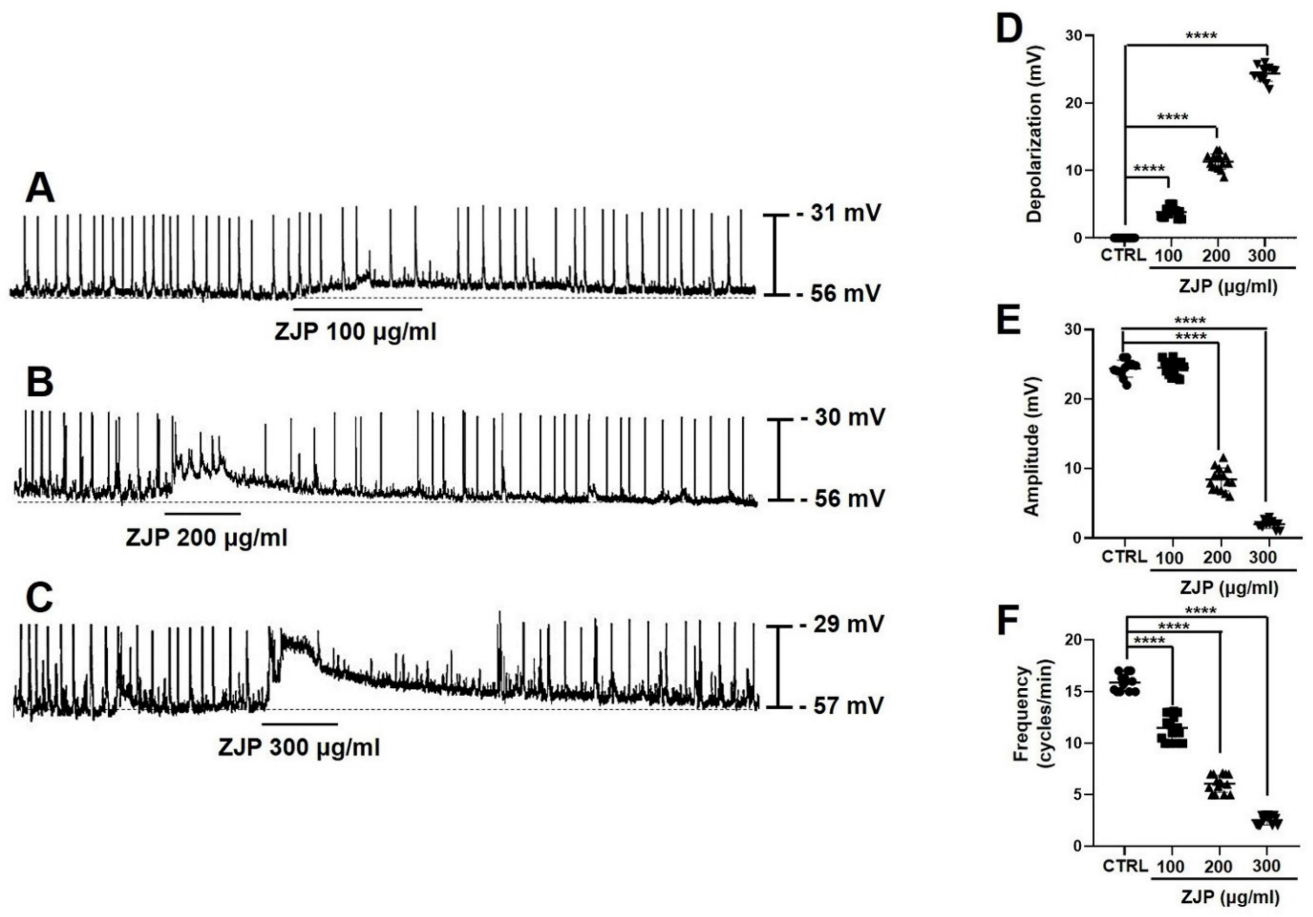
Effects of ZJP on ICCs membrane potential and pacemaker potentials. (A-C) ZJP-induced ICCs depolarization. (D-F) Dose-dependent effects of ZJP on membrane potential, amplitude, and frequency. All results shown as mean ± SE. ****p < 0.0001 vs. control cells. ZJP: Zuojin Pill. CTRL: control. ICCs: interstitial cells of Cajal.

Primary ICCs exhibited a spontaneous pacemaker potential depolarization of 24.2 ± 1.5 mV (mean amplitude ± SE) at a frequency of 15.8 ± 0.8 cycles/min from a mean resting membrane potential of -56.3 ± 3.2 mV (average of n = 48 cells) (Figure 2). Administration of ZJP (100-300 μg/mL) to the extracellular medium dose-dependently depolarized the membrane potential and reduced both pacemaker potential amplitude and frequency (Figure 2A-2C). Mean depolarization was 4.1 ± 0.6 mV at 100 μg/mL (n = 15; p < 0.0001), 10.9 ± 0.8 mV at 200 μg/mL (n = 15; p < 0.0001), and 24.9 ± 1.4 mV at 300 μg/mL (n = 13; p < 0.0001) (Figure 2D), while mean amplitude fell from 24.7 ± 1.2 mV at 100 μg/mL to 8.1 ± 1.9 mV at 200 μg/mL (p < 0.0001) and 1.6 ± 0.5 mV at 300 μg/mL (p < 0.0001) (Figure 2E), and mean frequency decreased to 11.6 ± 1.4 cycles/min at 100 μg/mL (p < 0.0001), 5.9 ± 0.9 cycles/min at 200 μg/mL (p < 0.0001), and 2.5 ± 0.5 cycles/min at 300 μg/mL (p < 0.0001) (Figure 2F). These results indicate that ZJP regulates ICCs pacemaker potential.

### Activation of 5-HT4 receptors contributes to ZJP-induced ICCs depolarization and modulation of pacemaker potentials

**Figure 3:**
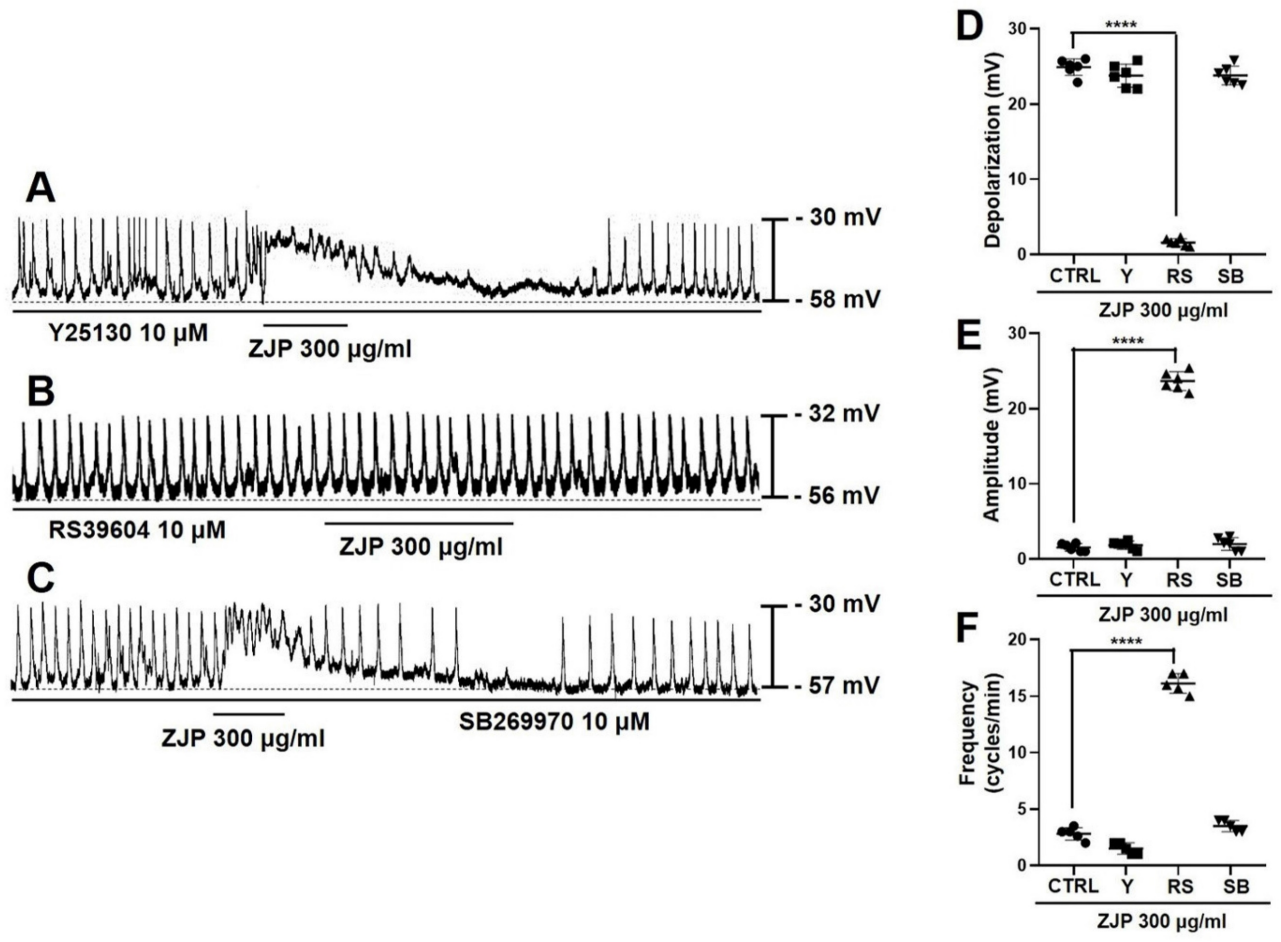
Contributions of 5-HT4 receptors to ZJP-induced ICCs depolarization and modulation of pacemaker potentials. (A-C) Responses to ZJP in the presence of Y25130 (A), RS39604 (B), or SB269970 (C). The effects of ZJP were blocked by the 5-HT4 receptor antagonist but not 5-HT3 or 5-HT7 antagonist. (D-F) Summary of antagonist effects on ZJP-induced depolarization, amplitude, and frequency. All results shown as mean ± SE. ****p < 0.0001 vs. ZJP alone. ZJP: Zuojin Pill. CTRL: Control. Y: Y25130. RS: RS39604. SB: SB269970. ICCs: interstitial cells of Cajal.

Serotonin receptor subtypes 5-HT3, 4, and 7 are expressed by ICCs 22-24, so we examined if the 5-HT3 receptor antagonist Y25130, 5-HT4 antagonist RS39604, and 5-HT7 antagonist SB269970 influenced ZJP-induced modulation of ICCs pacemaker potentials (Figure 3A-3C). Mean depolarization induced by 300 μg/mL ZJP was 23.9 ± 1.5 mV in Y25130 and 24.3 ± 1.4 mV in SB269970 but only 1.5 ± 0.6 mV in RS39604 (p < 0.0001 vs. ZJP alone) (Figure 3D), while mean amplitude was 1.8 ± 0.6 mV in Y25130 and 1.9 ± 1.0 mV in SB269970 but 23.5 ± 1.3 mV in RS39604 (p < 0.0001 vs. ZJP alone) (Figure 3E), and mean frequency was 1.5 ± 0.5 cycles/min in Y25130 and 3.5 ± 0.5 cycles/min in SB269970 but 16.1 ± 0.9 cycles/min in RS39604 (p < 0.0001 vs. ZJP alone) (Figure 3F). Thus, blockade of 5-HT4 receptors restored membrane potential, pacemaker amplitude, and pacemaker frequency to near control levels in the presence of ZJP.

### Activation of muscarine M3 receptors contributes to ZJP-induced ICCs depolarization and modulation of pacemaker potentials

**Figure 4:**
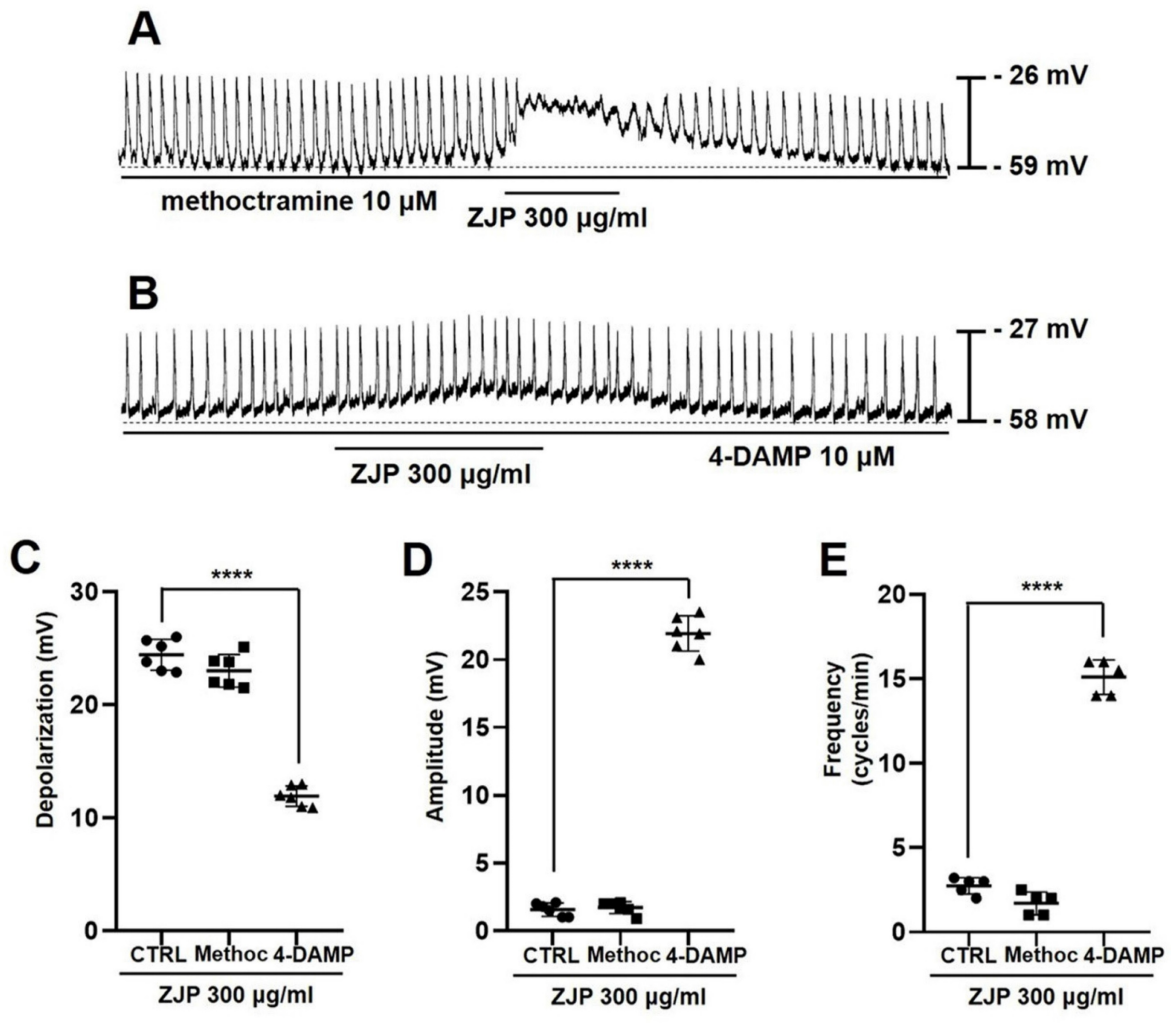
Contributions of muscarinic M3 receptors to ZJP-induced ICCs depolarization and modulation of pacemaker potentials. (A, B) Responses to ZJP in the presence of methoctramine (A) and 4-DAMP (B). Pretreatment with 4-DAMP but not methoctramine inhibited ZJP-induced ICCs depolarization. (C-E) Summary of antagonist effects on ZJP-induced depolarization, amplitude, and frequency. Results are expressed as mean ± SE. ****p < 0.0001. ZJP: Zuojin Pill. CTRL: Control. Methoc: Methoctramine. ICCs: interstitial cells of Cajal.

ICCs exclusively express muscarinic receptor subtypes M2 and M3 25-27, so we also examined the effects of the M2 antagonist methoctramine and M3 antagonist 4-DAMP on ZJP-induced changes in pacemaker potentials (Figure 4A and 4B). Mean depolarization was 23.6 ± 1.5 mV in methoctramine but only 12.2 ± 1.0 mV in 4-DAMP (p < 0.0001 vs. ZJP alone) (Figure 4C). Further, mean pacemaker potential amplitude was 1.7 ± 0.5 mV in ZJP plus methoctramine but 22.1 ± 1.4 mV in ZJP plus 4-DAMP (p < 0.0001 vs. ZJP alone) (Figure 4D), while mean frequency was 1.7 ± 0.7 cycles/min in methoctramine plus ZJP but 15.1 ± 1.0 cycles/min in ZJP plus 4-DAMP (p < 0.0001 vs. ZJP alone) (Figure 4E). Thus, M3 receptors also contribute to ZJP-induced effects on pacemaker potentials.

### G-protein activation is required for ZJP-induced depolarization of ICCs and pacemaker potential modulation

**Figure 5:**
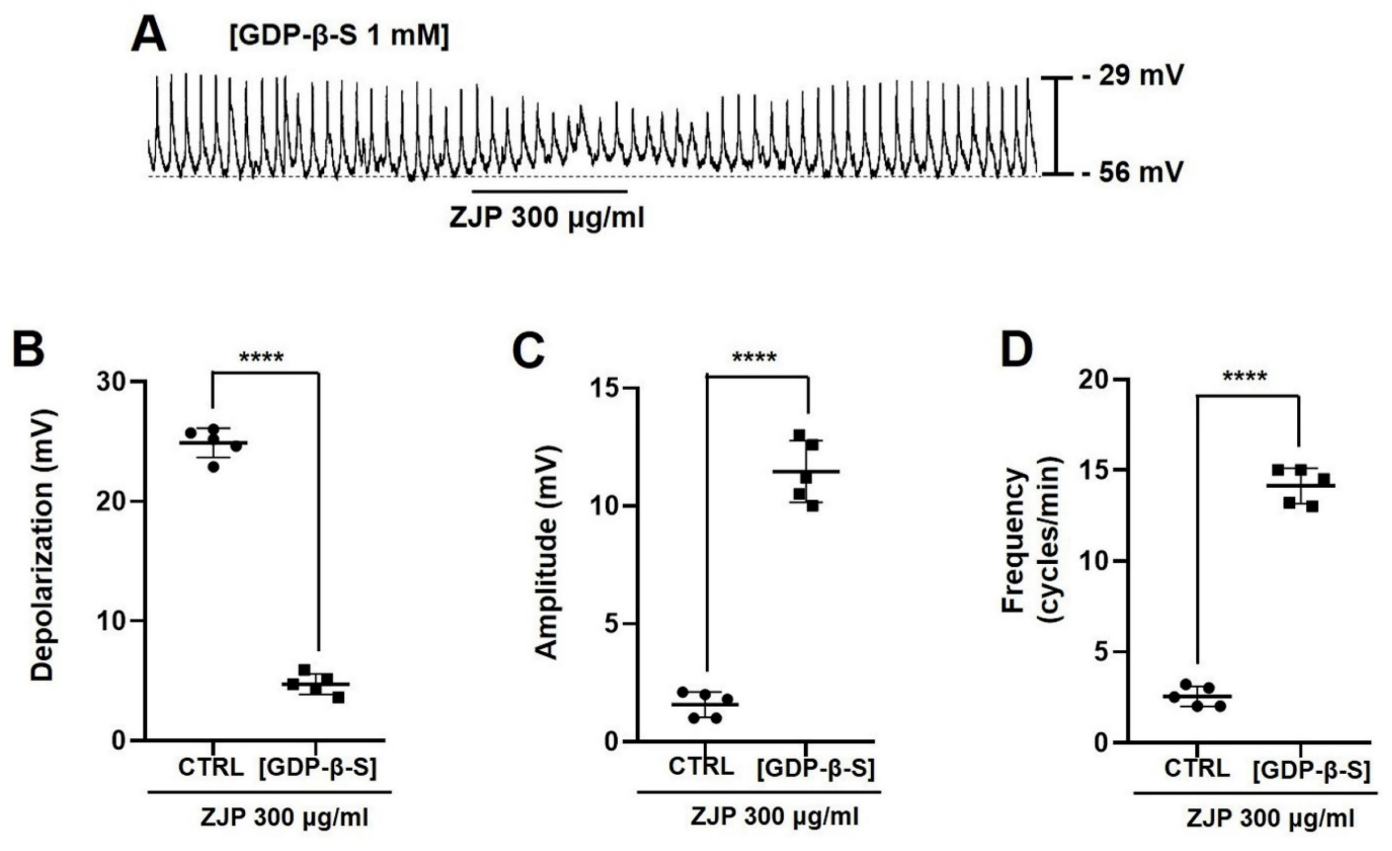
Requirement of G-protein transduction for ZJP-induced ICCs depolarization and modulation of pacemaker potentials. (A) Intracellular GDP-β-S blocked ZJP-induced depolarization. (B-D) Summary of GDP-β-S effects on ZJP-induced depolarization, amplitude, and frequency. Results are presented as mean ± SE. ****p < 0.0001. ZJP: Zuojin Pill. CTRL: Control. ICCs: interstitial cells of Cajal.

Inclusion of the G-protein inhibitor GDP-β-S (1 mM) in the whole-cell patch pipette (Figure 5A) reduced mean ZJP-induced depolarization to 4.7 ± 1.1 mV (Figure 5B), increased mean pacemaker potential amplitude to 11.5 ± 1.3 mV (Figure 5C), and increased mean pacemaker potential frequency to 14.1 ± 1.0 cycles/min (Figure 5D) (all p <0.0001 compared to ZJP alone). Restoration of these values to near control levels indicates that G-protein transduction is necessary for the effects of ZJP.

### Calcium influx and intracellular release are required for ZJP-induced ICCs depolarization

**Figure 6:**
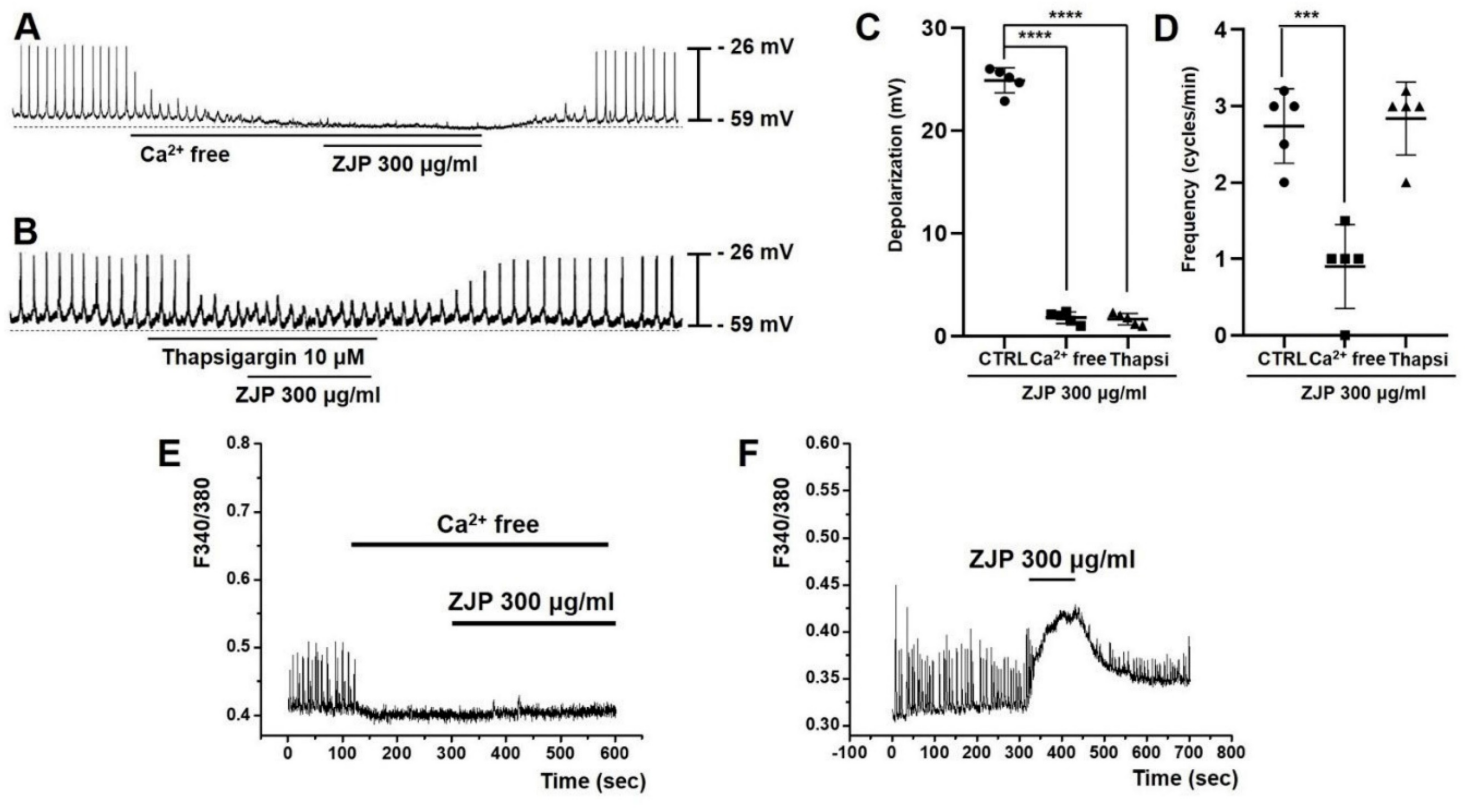
Requirement of intracellular Ca^2+^ signaling for ZJP-induced ICCs depolarization. (A and B) Abrogation of ZJP-induced depolarization in Ca^2+^-free medium (A) and following thapsigargin exposure (B). (C and D) Summary of changes in depolarization and frequency. (E and F) Spontaneous intracellular Ca^2+^ signals were eliminated by removal of extracellular Ca^2+^. (F) Spontaneous calcium changes were augmented by ZJP. Results presented as mean ± SE. ***p < 0.001. ****p < 0.0001. ZJP: Zuojin Pill. CTRL: Control. Thapsi: Thapsigargin. ICCs: interstitial cells of Cajal.

Both removal of extracellular calcium and administration of the Ca^2+^-ATPase inhibitor thapsigargin (which depletes intracellular calcium stores) inhibited ZJP-induced ICCs depolarization (Figure 6A and 6B). Mean ZJP-induced depolarization was 1.7 ± 0.5 mV in calcium-free medium and 1.6 ± 0.6 mV in thapsigargin (both p <0.0001 vs. ZJP alone) (Figure 6C). However, mean frequency was 0.9 ± 0.5 cycles/min in calcium-free medium (p < 0.001 vs. ZJP alone) and 2.8 ± 0.4 cycles/min in thapsigargin (Figure 6D). These results indicate that ZJP-induced ICCs depolarization is regulated by both intracellular and extracellular calcium.

### Intracellular free calcium elevations are required for ZJP-induced ICCs depolartization and modulation pacemaker potentials

Spontaneous calcium changes were observed in fura 2-loaded ICCs during pacemaker potentials. These calcium transients were markedly reduced by removal of extracellular calcium concomitant with abrogation of ZJP effects (Figure 6E) but increased by ZJP in the presence of extracellular calcium (Figure 6F).

### Phospholipase C (PLC) and inositol triphosphate (IP3) signaling pathways mediate ZJP-induced ICCs depolarization

**Figure 7:**
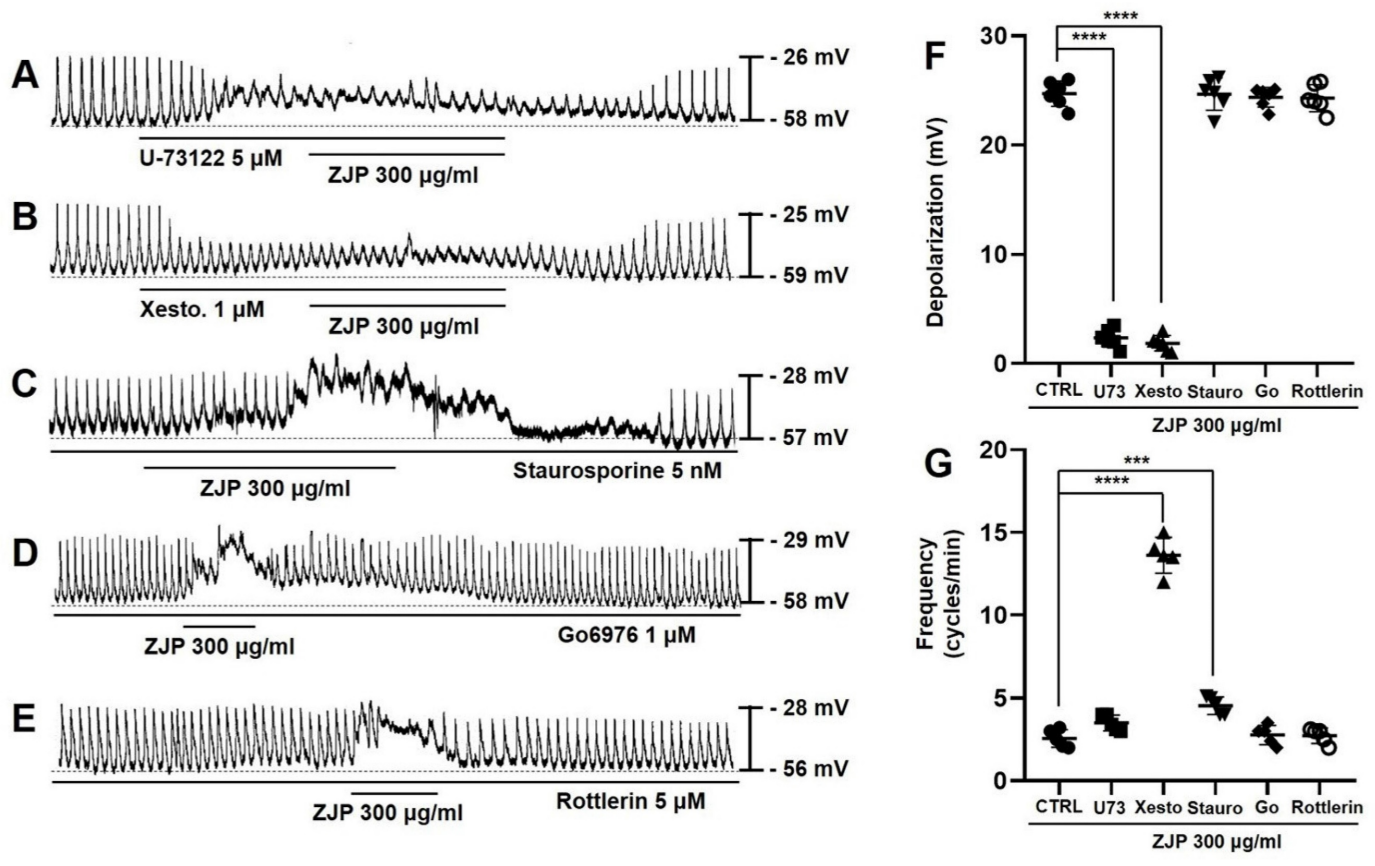
Requirement of phospholipase C, IP3, and PKC signaling for ZJP-induced ICCs depolarization and modulation of pacemaker potentials. (A and B) Both the PLC inhibitor U-73122 (A) and IP3 receptor inhibitor xestospongin C (B) blocked the effects of ZJP. (C, D, and E) The effects of ZJP were not blocked by the PKC inhibitor staurosporine (C), Go6976 (D), or rottlerin (E). (F and G) Summary of changes in depolarization and frequency. ***p < 0.001. ****p < 0.0001. ZJP: Zuojin Pill. CTRL: Control. U73: U-73122. Xesto: Xestospongin C. Stauro: Staurosporine. Go: Go6976. ICCs: interstitial cells of Cajal.

Zuojin Pill-induced ICCs depolarization and modulation of pacemaker potentials were also examined in cells pretreated with the PLC inhibitor U-73122, IP3 receptor blocker xestospongin C, the broad-spectrum PKC inhibitor staurosporine, calcium-dependent PKC α/β inhibitor Go6976, or calcium-independent PKC δ inhibitor rottlerin to assess the contributions of these downstream signaling pathways. Zuojin Pill-induced ICCs depolarization was inhibited in the presence of 5 μM U-73122 (Figure 7A) and 1 μM xestospongin C (Figure 7B), while robust depolarizations were still observed in the presence of 5 nM staurosporine (Figure 7C), 1 μM Go6976 (Figure 7D), and 5 μM rottlerin (Figure 7E). Specifically, mean depolarization was 2.3 ± 0.9 mV in U-73122 and 1.6 ± 0.5 mV in xestospongin C (both p <0.0001 vs. ZJP alone) but 24.6 ± 1.9 mV in staurosporine, 24.4 ± 1.1 mV in Go6976, and 24.1 ± 1.3 mV in rottlerin (Figure 7F). Moreover, mean pacemaker potential frequency was 3.5 ± 0.5 cycles/min in U-73122, 13.6 ± 1.1 cycles/min in xestospongin C (p < 0.0001), 4.5 ± 0.5 cycles/min in staurosporine (p < 0.001), 2.8 ± 0.6 cycles/min in Go6976, and 2.7 ± 0.5 cycles/min in rottlerin (Figure 7G). These results indicated that the PLC and IP3 pathways but not PKC pathways are involved in ZJP-induced ICCs depolarization and also modulate pacemaker activity.

### MAPK pathways mediate ZJP-induced ICCs depolarization and pacemaker potential modulation

**Figure 8:**
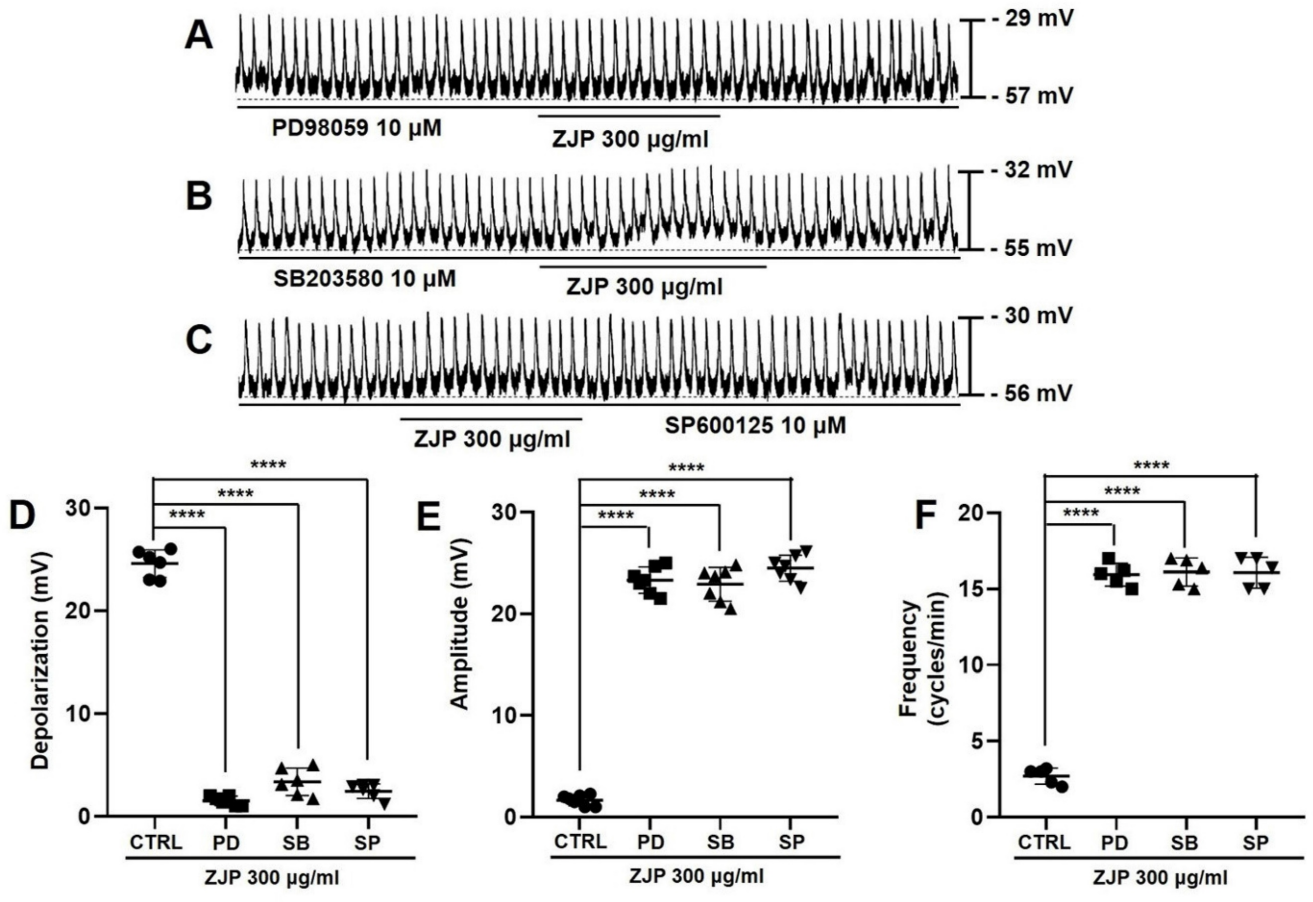
Requirement of MAPK signaling for ZJP-induced ICCs depolarization and modulation of pacemaker potentials. (A-C) The MAPK inhibitors PD98059 (A), SB203580 (B), and SP600125 (C) blocked ZJP-induced ICCs depolarization. (D-F) Summary of changes in depolarization, amplitude, and frequency. ****p < 0.0001. ZJP: Zuojin Pill. CTRL: Control. PD: PD98059. SB: SB203580. SP: SP600125. ICCs: interstitial cells of Cajal.

Zuojin Pill-induced ICCs depolarization and modulation of pacemaker potentials were also inhibited in the presence of the MAPK inhibitors PD98059, SB203580, and SP600125 (Figure 8A-8C). Mean depolarization was 1.4 ± 0.5 mV in PD98059, 3.1 ± 1.5 mV in SB203580, and 2.5 ± 0.9 mV in SP600125 (all p <0.0001 compared to the control condition) (Figure 8D), while mean pacemaker potential amplitude was 23.1 ± 1.4 mV in PD98059, 22.7 ± 1.7 mV in SB203580, and 24.5 ± 1.3 mV in SP600125 (all p <0.0001 vs. ZJP alone) (Figure 8E). Moreover, mean frequency was 15.9 ± 0.8 cycles/min in PD98059, 16.1 ± 0.9 cycles/min in SB203580, and 16.1 ± 1.0 cycles/min in SP600125 (p < 0.0001 compared to ZJP alone) (Figure 8F). These results indicate that the MAPK pathway is involved in ZJP-induced ICCs depolarization.

### ZJP restores normal ITR in GMD model mice

**Figure 9:**
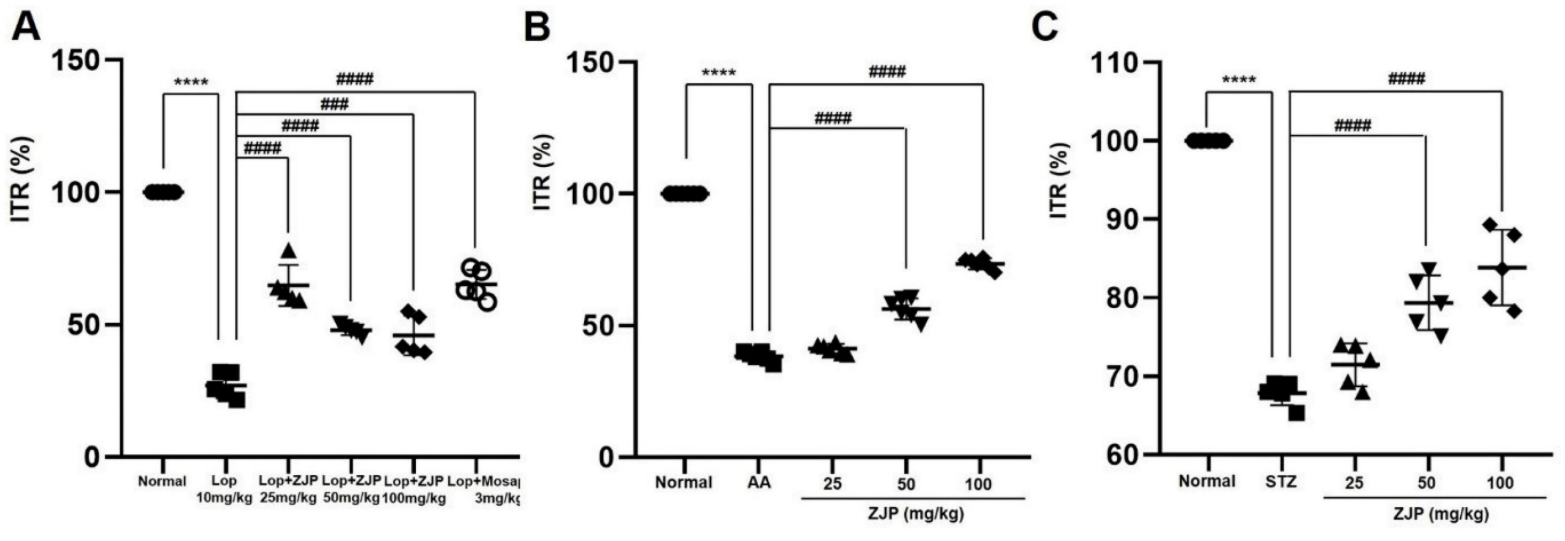
ZJP enhances intestinal transit rate in wild type mice and restores normal intestinal transit rate in GMD model mice. (A-C) ZJP increased ITR in wild type mice (A), and restored normal ITR in AA-induced GMD mice (B) and STZ-induced GMD mice (C). Results are presented as mean ± SE. ****p < 0.0001 vs. untreated wild type group. ###p < 0.001 and ####p < 0.0001 vs. loperamide-treated and control groups. ZJP: Zuojin Pill. CTRL: Control. Lop: Loperamide. AA: Acetic acid. STZ: Streptozotocin.

**Figure 10:**
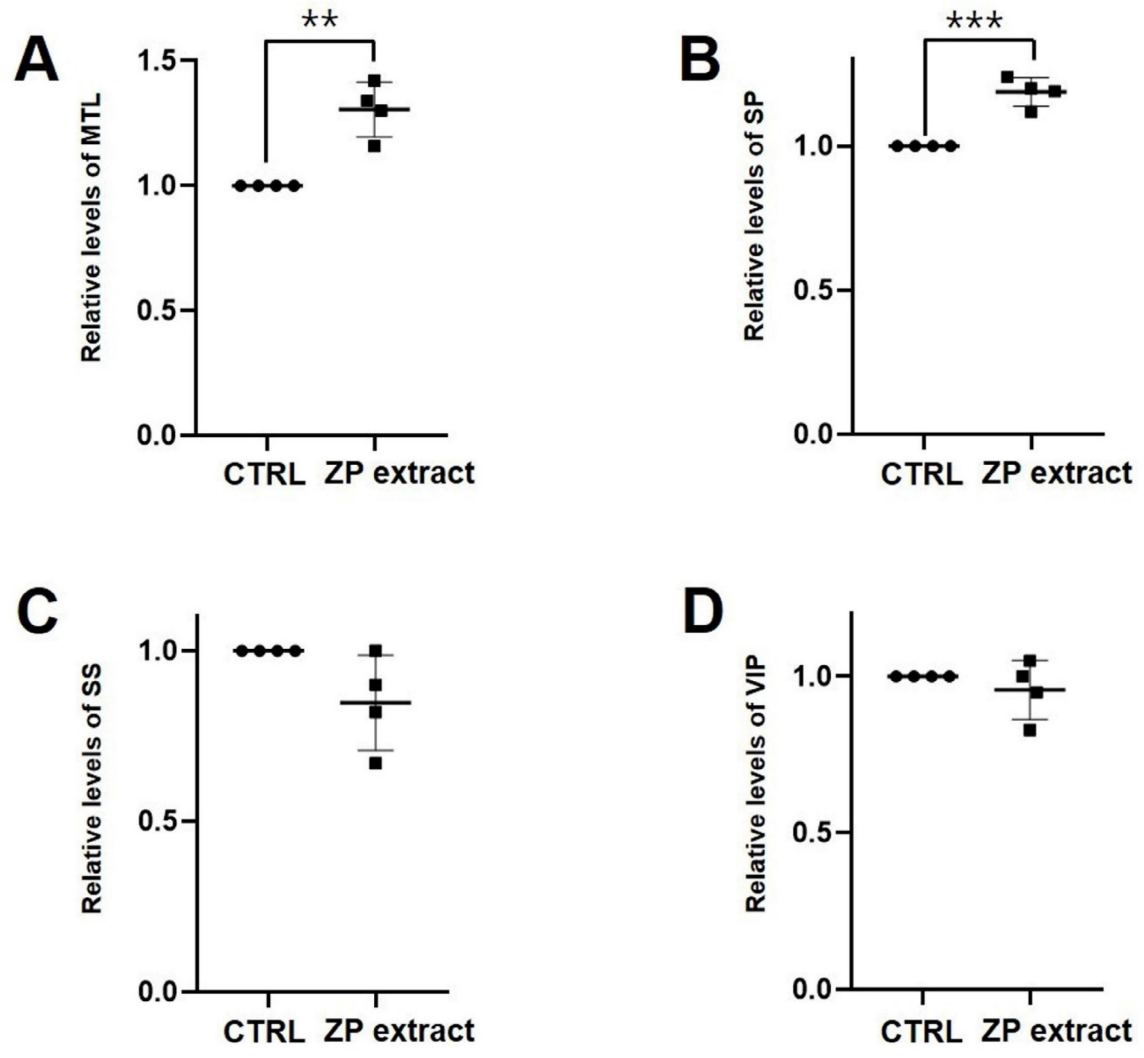
Impact of ZJP on gut hormones in wild type mice. (A and B) Serum MTL and SP levels were increased by ZJP. (C and D) SS and VIP levels remained unchanged. Results presented as mean ± SE. **p < 0.01. ***p < 0.001. ZJP: Zuojin Pill. CTRL: Control. MTL: Motilin. SP: Substance P. SS: Somatostatin. VIP: Vasoactive Intestinal Polypeptide. SE: SE.

As expected, loperamide reduced ITR in wild type ICR mice by 26.4% ± 5.3% (p < 0.0001 vs. untreated controls), while 25 mg/kg ZJP increased ITR by 65.8 ± 10.7% (p < 0.0001), 50 mg/kg by 47.6 ± 0.4% (p < 0.0001), and 100 mg/kg by 47.4% ± 10.9% (p < 0.001), roughly equivalent to the positive control mosapride (65.1% ± 9.3%, p < 0.0001; Figure 9A). Moreover, ZJP restored ITR in both AA and STZ mouse models of GMD (Figure 9B and 9C). In addition, ZJP markedly increased serum levels of hormones MTL and SP (Figure 10A and 10B), but not SS or VIP (Figure 10C and 10D). These results indicate that ZJP may increase ITR through MTL and SP signaling.

## Discussion

We demonstrate that the TCM preparation Zuojin Pill can modulate the pacemaker activity of ICCs and the intestinal transit rate in both wild type mice and gastric motility disorder model mice. These effects are mediated at least in part by 5-HT4 or M3 receptor activation and G-protein coupled induction of Ca^2+^, PLC, IP3, and MAPK signaling pathways. These findings on the complex molecular mechanisms underlying the therapeutic effects of ZJP provide a foundation for further development of ZJP-based GMD treatments.

Zuojin Pill has multiple bioactivities but is prescribed primarily for the treatment of GI disorders 1,2. The therapeutic effects of ZJP are associated with anti-inflammatory 3,11, anti-apoptotic 1,7,8, anti-bacterial 10-14, and mucosal protective properties 28, which are particularly efficacious for the treatment of chronic gastritis, colitis, and other forms of GI dysfunction caused by stress among other factors 3,6,11. In addition, ZJP is considered an excellent treatment for dyspepsia and inflammatory bowel disease (IBD) 10,11. Despite its widespread use and documented efficacy for a broad spectrum of GI disorders, there are no reports on the regulation of GI motility. In this study, we confirmed that ZJP regulates ICCs pacemaker potentials and increases ITR. Therefore, ZJP may regulate GI motility via ICCs in the GI tract. Future studies are warranted to elucidate the contribution of each herbal component and bioactive compound for promoting GI motility.

Serotonin and muscarinic receptors are known to regulate GI tract function, including motility 29,30. The GI tract expresses 5-HT3, 5-HT4, and 5-HT7 receptors 22-24. The G-protein-coupled receptor (GPCR) 5-HT3 is expressed primarily by GI sensory nerves and is associated with nausea and vomiting reflexes as well suppression of convulsant activity 31,32, while the GPCR 5-HT4 promotes GI motility and thus is activated therapeutically to treat constipation 33,34. Alternatively, 5-HT7 is associated with the relaxation of smooth muscles in the GI tract and blood vessels and may be a good target for the treatment of depression because of its involvement in mood regulation 35. Muscarinic receptors are activated by acetylcholine, a neurotransmitter of the parasympathetic nervous system. Muscarinic M2 and M3 receptors are the main subtypes expressed by GI ICCs 25,26. The M2 receptor is also expressed abundantly by smooth muscle cells and activation reduces cAMP concentration through the G-protein Gi, thereby promoting relaxation 36. In addition, M2 regulates inward-rectifying potassium channels 36, but this effector pathway did not appear to influence membrane potential or pacemaker potentials. The M3 receptor is also expressed by smooth muscles as well as gland cells and directly regulates GI motility and digestive fluid secretion by increasing intracellular calcium signaling via Gq 36. In this study, ZJP caused ICCs depolarization through 5-HT4 or M3 receptors (Figure 2,3,4 and 5). These receptors are thought to interact to promote GI motility, potentially through intracellular calcium regulation. Changes in calcium influx and intracellular release generate calcium oscillation that are essential for the rhythmic depolarization and repolarization of ICCs 37. These pacemaker potentials are in turn transmitted to the surrounding smooth muscle cells and enteric nerves, leading to the repeated contractions and relaxations required for peristalsis 37. In this study, removal of extracellular calcium or depletion of internal stores disrupted pacemaker potentials and modulation by ZJP (Figure 6). Moreover, these intracellular calcium oscillations were disrupted by removal of extracellular calcium, while ZJP enhanced intracellular calcium concentrations in the presence of extracellular calcium (Figure 6). Therefore, ZJP-induced ICCs depolarization and modulation of pacemaker activity is largely dependent on regulation of intracellular calcium.

**Figure 11:**
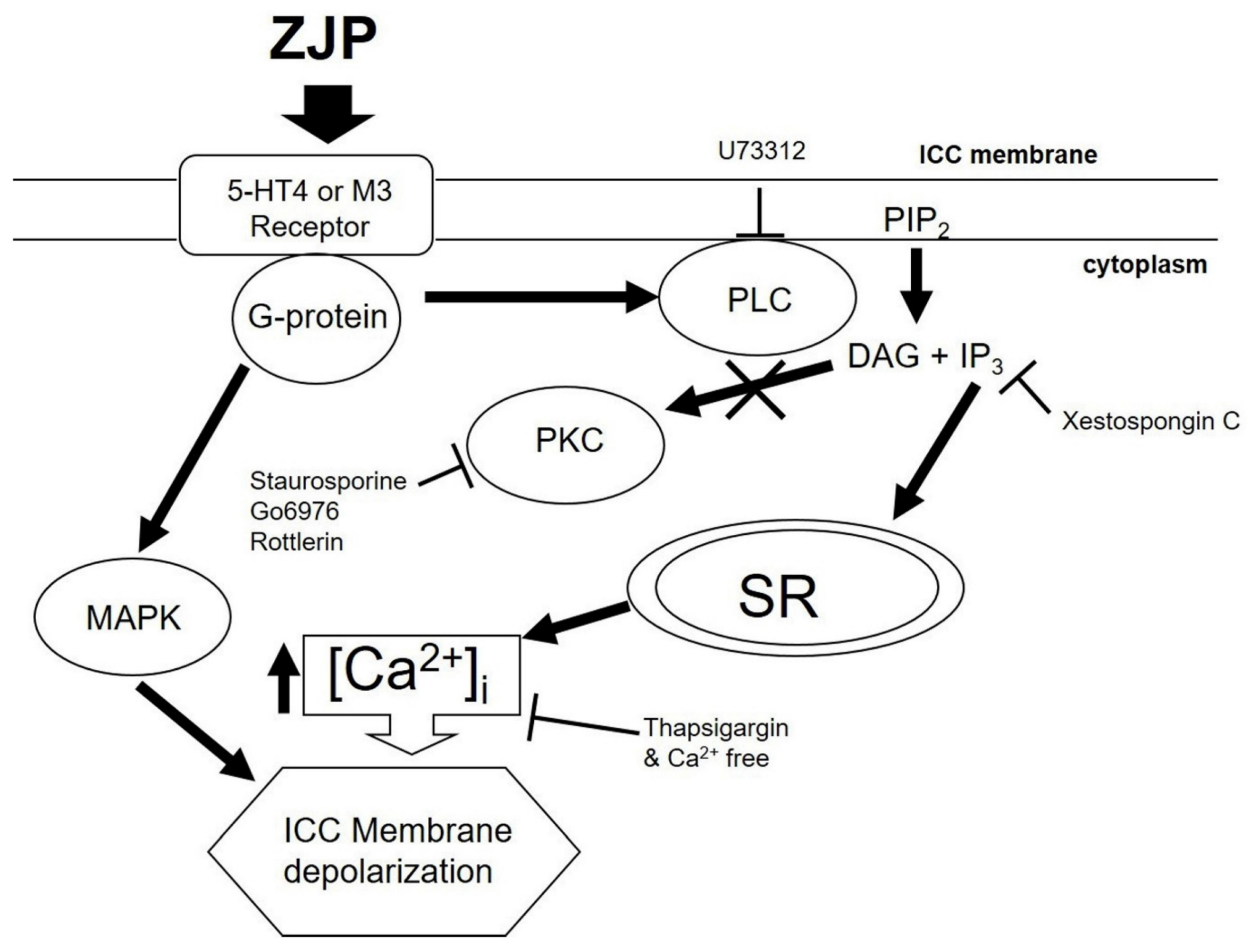
Schematic of signaling pathways mediating ZJP-induced ICCs depolarization and modulation of pacemaker potentials. ZJP-induced ICCs depolarization appears to be mediated by 5-HT4 and (or) M3 receptors and requires G-protein transduction and activation of MAPK, PLC-, IP3- and Ca^2+^-dependent signaling pathways. These signaling pathways have differential effects on ZJP-induced modulation of pacemaker potentials.

Intracellular calcium can activate and reciprocally be regulated by multiple downstream signaling pathways, including PLC, PKC, MAPK, and IP3 pathways, all of which are known to influence GI motility 38. Phospholipase C breaks down cell membrane phospholipids to produce two important signaling molecules, IP3 and diacylglycerol (DAG). While IP3 binds to the IP3 receptor in the sarcoplasmic reticulum to release stored calcium into the cytoplasm 39, DAG activates PKC, which in turn regulates GI motility by phosphorylating several protein substrates 40. The MAPK pathway regulates cell growth, differentiation, survival, and stress responses 41. In addition, it has been found to increase GI motility in normal and inflamed GI smooth muscles 42. In this study, PLC, IP3, and MAPK inhibitors blocked ZJP-induced ICCs depolarization and (or) pacemaker potential modulation, while PKC pathway inhibition had no such effects on ZJP responses (Figure 7 and 8). Activation of PLC and IP3 generation may induce ICCs depolarization by increasing intracellular calcium concentrations. In addition, MAPK may be directly involved in ICCs depolarization, although the target ionic channels remain unclear. In summary, these results show that ZJP depolarizes ICCs via multiple pathways, including 5-HT4 or M3 receptor activation and subsequent G-protein transduced activation of PLC, IP3, and MAPK pathways, which in turn modulate intracellular calcium release and extracellular influx (Figure 11).

ITR refers to the speed at which food passes through the GI tract under the influence of peristalsis. This motility process is regulated by numerous factors, among which intestinal hormones are essential for optimizing the digestive process and nutritional absorption by regulating smooth muscle contraction and secretion from glands and mucosal cells 43. As shown in Figures 9 and 10, ZJP increased the ITR as well as serum concentrations of promotility hormones MTL and SP. Therefore, ZJP likely regulates ITR by increasing the levels of MTL and SP hormones.

The long-term use of ZJP helps regulate stomach acid secretion, relieving symptoms like heartburn and gastritis caused by excessive acid 1,2,12-14. It is also effective for improving indigestion and bloating 1,2,6, and promoting digestion 10,11. In addition, ZJP can help reduce inflammation, providing long-term relief from chronic gastritis and gastric ulcers 10-14. In this study, experimental mice were administered ZJP by gavage at a range of doses (1, 2, or 5 g/kg) for 2 weeks to assess both efficacy and side effects profile. Over the course of two weeks, there were no significant changes in animal body weight. On the last day of the 2-week period, vital organs and tissues also appeared normal by gross inspection. Therefore, ZJP may be safe for long-term use at 5 g/kg. Treatment for 2 weeks also reversed ITR suppression caused by loperamide, indicating that ZJP increases intestinal motility within physiological ranges, and there were no notable changes in stool consistency.

Research on ICCs is critical for a better understanding of GI motility control. As ICCs coordinate the electrical activities of the GI tract and facilitate the digestion and movement of food 15, they are key targets for treating GMDs. Indeed, ICCs dysfunction is associated with several GI diseases 44. For example, damage or deterioration of ICCs can cause GI tract dysfunction, intestinal obstruction, irritable bowel syndrome, and diabetic GI paralysis 44. Therefore, understanding the role of ICCs in ITR is essential for elucidating the pathophysiology of these diseases and may provide important clues for the development of new treatments. For example, drugs that improve or protect ICCs function may effectively treat GMD. Therefore, our ICCs research contributes to understanding GI disease pathology and treatment development. When ICCs undergo depolarization, they significantly impact both the muscle tone and phasic contractions of the GI muscles 45,46. Depolarization of ICCs leads to the transmission of electrical signals to the smooth muscle cells, causing their membrane potential to also depolarize. This, in turn, can increase the muscle tone, meaning the smooth muscles may shift from a relaxed state to a more contracted state. In other words, ICCs depolarization can raise the baseline tension (tone) of the GI muscles, causing them to remain in a more contracted state. Additionally, when ICCs depolarization occurs, the amplitude and frequency of slow waves may increase, leading to more frequent phasic contractions. This heightened activity results in stronger and more frequent contractions of the GI muscles, enhancing the movement of contents through the digestive tract via peristalsis. Consequently, this could also increase ITR. ICCs are also closely connected with the enteric nervous system (ENS) 45,47. ICCs are in close contact with nerve endings of the ENS and can receive neural signals. Both excitatory and inhibitory neurotransmitters from the ENS can act on ICCs. Excitatory neurotransmitters, such as acetylcholine, act through ICCs to stimulate contraction of the GI smooth muscle, while inhibitory neurotransmitters, like nitric oxide (NO), inhibit ICCs activity, promoting relaxation of the smooth muscle. ICCs also transmit electrical signals from the ENS to the GI smooth muscle. These signals, combined with slow waves generated by the ICCs, regulate phasic contractions in the GI muscles. In this way, neural signals are relayed through ICCs to induce more coordinated contractions and relaxations of the muscles. Furthermore, ICCs and ENS interact to regulate the rhythm and strength of GI motility. Neural signals modulate ICCs activity, which in turn fine-tunes the movement of the smooth muscle. For example, during digestion, excitatory neural signals stimulate ICCs, enhancing muscle contractions for efficient processing of food.

TCM is based on centuries of accumulated experience and knowledge 48 and is preferred by many as it relies on natural herbal ingredients that generally lead to fewer side effects compared to artificial chemicals. These preparations not only alleviate symptoms but help restore overall balance in the body and enhance immunity, promoting better health. Additionally, TCMs often demonstrates gradual effects and are less likely to cause sudden changes or adverse reactions. When used alongside modern medicine, TCMs can enhance treatment efficacy and minimize side effects. For instance, ZJP has been reported to have fewer side effects than conventional antidepressants and to reduce GI side effects caused by the antidepressant venlafaxine 49. The current study further supports the use of TCM as primary or adjunct treatment and may enhance trust in this medicinal system.

In conclusion, the present study shows that ZJP can depolarize ICCs pacemaker potentials via 5-HT4 or M3 receptor activation and downstream induction of PLC, IP3, MAPK, and intracellular signaling pathways. Additionally, ZJP increased ITR, likely by triggering the release of MTL and SP. Therefore, the present study supports the use of ZJP for GMD treatment. However, efficacy in the human GI tract has not been confirmed, and the specific bioactive components have not been identified. In the future, we plan to conduct further in-depth research on this topic.

## Figures and Tables

**Table 1:** Analytic conditions for detection of evodiamine, limonin, rutaecarpine, berberine, and palmatine in Zuojin Pill.

Time (minute)	0.1% FA/water (%)	0.1% FA/acetonitrile (%)	Flow rate (ml/minute)
0	98	2	0.40
1.5	98	2	0.40
2.0	90	10	0.40
3.0	75	25	0.40
5.0	75	25	0.40
6.0	70	30	0.40
8.0	60	40	0.40
9.0	50	50	0.40
10.0	50	50	0.40
11.0	40	60	0.40
12.0	2	98	0.40
14.0	98	2	0.40
16.0	98	2	0.40

**Table 2:** Concentrations of evodiamine, limonin, rutaecarpine, berberine, and palmatine in the Zuojin Pill sample used for experiments as measured by UPLC

		Content. ppm	
Evodiamine		19.186 ± 0.105	
Limonin		0.906 ± 0.026	
Rutaecarpine		18.082 ± 0.295	
Berberine		872.077 ± 5.391	
Palmatine		8.893 ± 0.025	
